# Stability of Antiradical Activity of Protein Extracts and Hydrolysates from Dry-Cured Pork Loins with Probiotic Strains of LAB

**DOI:** 10.3390/nu10040521

**Published:** 2018-04-22

**Authors:** Paulina Kęska, Joanna Stadnik

**Affiliations:** Department of Animal Raw Materials Technology, Faculty of Food Science and Biotechnology, University of Life Sciences in Lublin, Skromna 8, 20-704 Lublin, Poland; paulina.keska@up.lublin.pl

**Keywords:** antiradical peptides, dry-cured meat products, in vitro digestion

## Abstract

The application of starter cultures to improve quality and safety has become a very common practice in the meat industry. Probiotic strains of lactic acid bacteria (LAB) can also bring health benefits by releasing bioactive peptides. The aim of this work was to evaluate the stability of antiradical activity of protein extracts from LAB-inoculated dry-cured pork loins during long-term aging and evaluate their hydrolysates after simulated gastrointestinal digestion. Analyses of hydrolysates by using liquid chromatography-tandem mass spectrometry (LC-MS/MS) were strengthened with in silico analysis. The highest antiradical activity of the protein extracts was observed after 180 days of aging. The influence of the strain used (LOCK, BAUER, or BB12) on the inactivation ability of ABTS radicals varied during long-term aging. The IC_50_ values indicated the higher antiradical properties of salt-soluble (SSF) compared to water-soluble fraction (WSF) of proteins. The peptides generated by in vitro digestion have MW between 700 and 4232 Da and their length ranged from 5 to 47 amino acids in a sequence where Leu, Pro, Lys, Glu, and His had the largest share. This study demonstrates that the degradation of pork muscle proteins during gastrointestinal digestion may give rise to a wide variety of peptides with antiradical properties.

## 1. Introduction

During the last several decades, the application of starter cultures has become a common practice in the production of more consistent and stable fermented meat products in order to improve their quality and safety, reduce variability, and enhance sensory characteristics. The starter groups used in meat fermentation are, by order of importance, lactic acid bacteria (LAB) (mainly *Lactobacillus* spp. and *Pediococcus* spp.), nonpathogenic coagulase-negative staphylococci (primarily *Staphylococcus* spp. and *Kocuria* spp.), molds (*Penicillium*), and yeasts (*Debaryomyces*) [1,2]. LAB play a significant role in meat fermentation by creating unfavorable conditions for pathogens and spoilage microorganisms via several mechanisms of action (e.g., competition for nutrients and living place on the product) or the production of substances inhibiting their growth especially lactic acid and/or acetic acid, acetoin, diacetyl, hydrogen peroxide, and bacteriocins. This contributes to product stability and safety [3,4]. The production of lactic acid also has a direct impact on the sensory properties of the product by providing a mild acidic taste and by supporting the drying process, which requires a sufficient decline in pH. Moreover, LAB influence the sensory characteristics of the fermented meats by producing small amounts of acetic acid, ethanol, acetoin, carbon dioxide, pyruvic acid, and their ability to initiate the production of aromatic compounds from proteinaceous precursors. Microorganisms other than LAB involved in meat fermentation mainly bring about and stabilize the desired sensory properties [5].

Use of probiotic starter cultures for the production of fermented meat products has attracted attention in recent years [6]. Probiotic LAB strains, in addition to shaping the technological and sensory characteristics of the product, can also bring health benefits [7]. Recent studies involving the use of LAB strains with probiotic properties, i.e., *Lactobacillus rhamnosus* LOCK900 (LOCK), *Bifidobacterium animalis* spp. *lactis* BB12 (BB12) and a potentially probiotic strain *Lactobacillus acidophilus* Bauer Ł0938 (BAUER) have confirmed the suitability of using them in dry-cured meat product formulations, which underlines their favorable effects on hygienic quality and sensory characteristics of the products [8,9]. Regardless of the health benefits of LAB bacteria strains resulting due to their probiotic character, the proteolytic system of LAB is very efficient in releasing bioactive peptides from food proteins [10,11]. Meat protein-derived bioactive peptides are promising candidates for ingredients of functional meat products [12]. Bioactive peptides are inactive in the parent protein sequence until they are released by enzymatic hydrolysis. This process occurs naturally within the gastrointestinal tract during normal metabolism of dietary proteins. The same process happens during fermentation or aging in meat processing [13,14]. Previous in silico studies have shown that pork meat has great potential for influencing the physiological functions of the body as a source of peptides with biological activities such as antioxidative or angiotensin I-converting enzyme (ACE) inhibitory properties as well as dipeptidyl peptidase IV (DPP-4) inhibitors [15]. Bioactive peptides exhibiting ACE inhibitory activity have been found after in vitro digestion of Spanish dry-cured ham by gastrointestinal proteases [16]. Moreover, the in vivo antihypertensive activity of bioactive peptides of dry-cured ham has been reported in animal models of hypertension [17]. Recent studies suggest that dry-cured ham rich in bioactive peptides may exert a plethora of activities over the cardiovascular system including lipid and glucose metabolism in healthy subjects with pre-hypertension [18]. 

Our preceding study [19] demonstrated that inoculation with the above mentioned probiotic or potentially probiotic strains of LAB influenced the antioxidant activity of peptides isolated from dry-cured pork loins. Since aging time and gastrointestinal digestion influence the activity of bioactive peptides, the study of the antiradical activity during aging and after in vitro gastrointestinal digestion would be of interest. Consequently, the aim of this study was to evaluate the stability of antiradical activity (by ABTS assay) of protein extracts from LAB-inoculated dry-cured pork loins during long-term aging and evaluate their hydrolysates after simulated gastrointestinal digestion with pepsin and pancreatin. Analyses of hydrolysates with the highest antiradical properties using liquid chromatography-tandem mass spectrometry (LC-MS/MS) were strengthened by in silico analysis.

## 2. Results and Discussion

The significance levels of the factors included in the experiment and obtained by the ANOVA are presented in Table 1. Treatment (inoculation), aging time, and the interaction between them showed a significant effect on proteolytic changes expressed as primary amino groups (-NH_2_) and antiradical activity (ABTS) before and after each step of in vitro gastrointestinal digestion and simulated absorption of dry-cured loins.

### 2.1. Stability of Antiradical Activity during Long-Term Aging

The highest antiradical activity against ABTS (%) was noted with the protein fractions extracted from dry-cured loins after 180 days of aging (see Table 2). However, the influence of either the strain used (LOCK, BAUER, or BB12) or the aging time on the inactivation ability of radicals generated from ABTS was ambiguous. Generally, the increase of antiradical activity of protein extracts up to 180 days of aging (expressed as percentage scavenging activity) was observed and followed by a systematic decrease during further aging stages. As far as WSF is concerned, at the first sampling point (after 28 days of aging), the highest antiradical activity (*p* < 0.05) was noted for the LOCK sample (69.94%).

However, in the subsequent steps (90, 180, and 270 days), the *Lactobacillus rhamnosus* LOCK900 strain was less effective in generating antiradical components with no statistically significant differences between LOCK and C sample. The highest antiradical effects were achieved for loins with BAUER after 90 and 180 days of aging (77.53% and 83.89%, respectively) and BB12 in 270 days (74.12%). The varied effect of probiotic LAB strains on generating antiradical molecules during long-term aging was also observed for SSF. Lower (*p* < 0.05) antiradical values were observed for batches with LAB compared with the variant that underwent spontaneous fermentation (C) at day 28. After this time, the behavior of this parameter in inoculated samples was ambiguous during the rest of the aging period. The biggest differences in antiradical activity (%) were noted between the sample subjected to spontaneous fermentation (C) and BB12 after 28, 90, 180, and 270 days. The LOCK batches had statistically significantly lower antiradical properties (%) compared to C (*p* < 0.05) at 28, 270, and 360 days. BAUER batches had significantly lower values (*p* < 0.05) of radical scavenging activity after 28, 180, and 360 days of aging as compared to C batches.

Antiradical properties were also defined as the concentration of the sample required to inhibit 50% of the radical-scavenging effect (IC_50_). Generally, the IC_50_ values clearly indicated the higher antiradical properties of protein-released components during long-term aging from SSF compared to WSF (see Table 2). With respect to the WSF, the antiradical activity of the hydrolysis products was stable between 28 and 180 day of aging (*p* > 0.05) and then decreased in all samples. SSF fractions were characterized by greater fluctuations of this parameter especially between 28 and 180 days of aging (*p* < 0.05).

Quantitative analysis was also used for investigating the correlation between antiradical activities and the content of protein degradation products in both fractions of muscle proteins. The correlation between the antiradical activity (expressed as percent inhibition and 1/IC_50_ (not IC_50_) showing parallelism with antiradical activity) and the primary amino groups content (µM/mL) was therefore determined [20].

As shown in Table 3, there was no positive correlation between the content of protein degradation products and the antiradical activity, which corresponds with other studies [21,22,23]. However, the strong negative correlation between the antiradical activity of the WSF fraction (expressed as 1/IC_50_) and the content of components with potential antiradical properties indicated the loss of bioactive properties during aging, which corresponds with the results presented in Table 1.

Therefore, the progressive degradation of proteins by endogenous meat enzymes and exogenous microbial proteases during 360 days of aging results in the disappearance of biological activity of protein-related compounds at a later stage. This may be a result of an overly extensive hydrolysis of the peptide chains [23].

### 2.2. Stability of Antiradical Activity during In Vitro Digestion

Determining the in vitro bioactivity to promote the beneficial effects of bioactive compounds should be carried out in the context of their immunity to digestive enzymes and to estimate their nutritional importance. In this context, hydrolysis of protein extracts obtained from dry-cured pork loin using gastrointestinal enzymes was accomplished using a two-step hydrolysis reaction. The first step was hydrolysis by pepsin (pH 2 at 37 °C for 2 hours) while the second step was the successive hydrolysis by pancreatin (pH 7 at 37 °C for 3 hours). The sequential digestion with pepsin and pancreatin provides a suitable model for evaluating peptides released in the intestinal tract. The effect of gastric in vitro (pepsin) and consecutive intestinal in vitro (pancreatin) digestion on protein extracts of dry-cured loins was discussed. The results are summarized in Table 4 and Table 5.

As noted earlier, the antiradical activity defined as percent inhibition and IC_50_ values were uncorrelated. However, the higher antiradical activity (%) was determined for the WSF fraction, which was probably indirectly related to a higher content of primary amino groups. The IC_50_ values unambiguously indicate SSF as a source of antiradical components and this tendency was maintained during in vitro gastrointestinal digestion (see Table 4 and Table 5) and simulated adsorption (see Table 6).

The decrease in the antiradical activity of the dry-cured loin protein hydrolysates determined by the ABTS test (expressed as % and IC_50_) was observed after gastric digestion (see Table 4) and compared to the undigested samples, which was followed by an increase of biological activity after pancreatin treatment (see Table 5). This observation corresponds with other authors’ findings [24,25,26,27]. The highest biological activity was achieved after 90 days of aging due to the hydrolytic degradation of proteins under the action of pepsin. During this period, an average of 83.60% was reported for WSF with significantly higher (*p* < 0.05) values of biological activity obtained for BB12 batches (86.51%). With regard to SSF, the antiradical activity after 90 days of aging was at an average level of 69.34% for C and LOCK (*p* > 0.05), while BAUER and BB12 batches was statistically significantly (*p* < 0.05) higher (70.61% and 75.18%, respectively). Pancreatin digested samples (see Table 5) showed values ranging from 85.94 for BAUER (28 days) to 98.56 for BB12 (90 days) for WSF and 72.45 for BAUER (28 days) to 95.86% for C (270 days) in the case of SSF. This suggests that fewer peptides with antiradical properties are associated with pepsin digestion than pancreatin. This may be due to the nature of the enzymes used. While pepsin can break down proteins and large peptides into smaller fragments by shielding the cleavage sites for further enzymes, pancreatin can primarily hydrolyze some of the peptides in smaller peptides and possibly amino acids. Pancreatin contains many enzymes including trypsin and additional proteases that give rise to hydrolysis activity, which leads to deeper breakdown of peptide chains. These results can be explained by the formation of a greater proportion of peptides and amino acids with hydrophilic properties during pancreatic digestion. Moreover, Zhu et al. [28] reported that pepsin cleaves peptides into smaller fragments, which exposes the internal groups to the environment. While trypsin hydrolyzed peptides into smaller chains, it also produced more free amino acids due to its greater hydrolytic activity. Therefore, these amino acids have greater affinity with water. This is because the increase of hydrophobic properties of GI digestion after pepsin treatment makes them less likely to react with the water-soluble ABTS radical. However, the increase of the hydrophilic property of GI digestion after pancreatin treatment favors their trapping of the ABTS radical [24].

After in vitro gastrointestinal digestion and simulated absorption, the antiradical properties of the hydrolysates increased compared to undigested proteins (see Table 6).

As expected, the best antiradical properties were achieved after in vitro gastrointestinal digestion and simulated adsorption of WSF extracted after 28 days of aging. These results are not consistent with the radical scavenging activity expressed by the IC_50_ for which the increase in antiradical properties attain the lowest biological activity (highest antiradical activity) on day 180, which was followed by a systematic decline until the end of the aging period was noted. This tendency was described earlier (see Table 2). In this period (180 days of aging), the statistically significantly higher biological activity as an antiradical within the WSF (*p* < 0.05) was shown for the BAUER and BB12 (IC_50_ = 0.02 µM/mL both; Table 6). With regard to SSF, the best antiradical properties (determined by % and IC_50_) were recorded after simulated absorption of the digested product after 180 days of aging with the best properties reported (*p* < 0.05) for the spontaneous fermentation (C) and BB12 batches. However, the differences between them were not statistically significant (*p* > 0.05).

### 2.3. Stability of Antiradical Activity during In Vitro Digestion

Dry-cured meats constitute a specific group of products in which the proteolytic processes take place from raw material to finished product, which can take up to 24 months. Proteolytic degradation of proteins takes place through exogenous enzymes of meat as well as by exogenous enzymes derived from microorganisms primarily responsible for fermentation processes that occur on raw meat. The proteolytic activity attributed to bacteria is characteristic of a particular strain. Therefore, different peptide and amino acid profiles are predicted depending on the LAB strains used during production. These aspects are crucial for detecting the specific functions of potentially bioactive peptides especially when digested with enzymes of the human gastrointestinal tract. The digestive tract affects the release of peptides from parent proteins and modifies or degrades peptides that may exhibit antiradical properties. In fact, the specificity of the enzyme affects the amount, size, and composition of the peptides, which influences the biological activity of the digested samples [25,29] and the degree of their absorption through the intestinal membrane.

Therefore, LC-MS/MS analysis was used to evaluate peptides (28, 90, and 180 days) and compare the peptide profile of the samples after in vitro digestion and absorption in the simulated gastrointestinal tract. Peptide sequences were identified and characterized by nano-LC-MS, which confirms the identification by exact mass determination with LTQ-Orbitrap. The cleavage of peptide bonds by digestive proteases leads to the release of peptides that have different lengths and free amino acids. The most typical well-known bioactive peptides are 200–1700 Da with fragment lengths from 2 to 14 amino acids so that they are able to easily pass through the gut lane and are capable of secreting nutritional value and bioactive functions [30].

**Figure 1:**
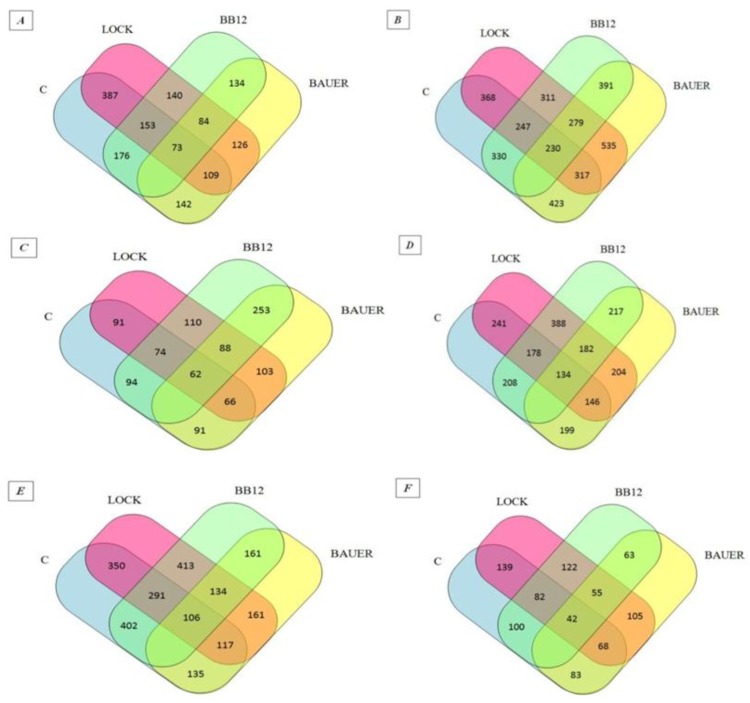
Venn diagrams of peptides obtained from dry-cured pork loins after digestion and simulated absorption: (**A**) peptides obtained from WSF after 28 days of aging, (**B**) peptides obtained from SSF after 28 days of aging, (**C**) peptides obtained from WSF after 90 days of aging, (**D**) peptides obtained from SSF after 90 days of aging, (**E**) peptides obtained from WSF after 180 days of aging, (**F**) peptides obtained from SSF after 180 days of aging. LOCK, sample inoculated with *Lactobacillus rhamnosus* LOCK900; BAUER, sample inoculated with *Lactobacillus acidophilus* Bauer Ł0938; BB12, sample inoculated with *Bifidobacterium animalis* ssp. *lactis* BB-12.

The peptides found in the digested samples have MW between 700 and 4232 Da and the length of the peptide fragments determined in the study ranged from 5 to 47 amino acids in a sequence, which is in agreement with other peptide profile studies obtained by digestion [31,32]. This probably indicates that they may have biological effects. The influence of individual strains on the peptide profile of the analyzed samples after the digestion and absorption process was summarized in the Venn diagram (see Figure 1) [33]. Venn diagrams indicated an increase in the number of peptides identified as common for the WSF of batches inoculated with LAB (from 84 to 134 sequences obtained after in vitro digestion and adsorption. At the same time in relation to the SSF, a decrease from 279 to 55 common sequences has been noted. After 180 days of aging, 402 common peptide sequences were identified for the WSF of C and BB12 samples while only 135 identified peptides were common for the C and BAUER batches. By analogy, taking into account the SSF, the C sample had more common peptide sequences with the LOCK (139) and less when compared to the BAUER sample (83). 

Due to the large number of peptides and, taking into account the highest antiradical activity of protein-released components extracted from the loins after 180 days of aging, the peptides derived from digestion and simulated absorption were identified by chromatographic methods and in silico analysis. The analyses were repeated twice and identical peptide sequences were selected for further analysis. In total, all peptides up to 480 sequences (38.53%) showed potency as antioxidants in the in silico study. In addition, the selected sequences were evaluated by rating capacity for bioactivity.

The peptide sequences with the highest *A* parameter (i.e., above 0.4) are presented in Table 7. Both sarcoplasmic and myofibrillar proteins have been described as precursors of bioactive peptides on the basis of in vitro assays. The amino acid composition, conformation, and hydrophobicity is correlated with antioxidant activity and likely determines the mechanism (transfer of hydrogen (HAT) or single electron (SET)) and the effectiveness of antioxidants [34,35]. It has been found that the peptides possibly containing substances and donating electrons are likely to react with the free radicals to terminate a radical reaction. Cys and Met residues, which contain nucleophilic sulphur side chains as well as Trp, Tyr, and Phe, have aromatic side chains and readily donate hydrogen atoms [28]. The antioxidant activity of peptides with one or more residues of His, Pro, Cys, Tyr, Trp, Phe, or Met and the presence of hydrophobic amino acids might be enhanced [36]. Peptides containing the amino acid residues Val, Leu, Ile, Ala, Phe, Lys, or Cys at the N-terminal and Trp, Tyr, His, or Pro in the sequence had been reported to show antioxidant activity [36,37]. This is caused by acidic or basic amino acid residues (Asp, Glu, His, Arg, or Lys) or hydrophilic amino acids (Ser, Thr) in this position. Moreover, as reported by Power et al. [35], it is suggested that a second amino acid adhering to the C-terminal is a major factor influencing antioxidant activity. If this amino acid has a high hydrogen bond and steric and low hydrophobicity, it will increase its anti-oxidative potential.

Within the peptide sequences identified in the present study (see Table 7), Leu (19.01%), Pro (15.85%), Lys (11.62%), Glu (7.39%), and His (7.04%) had the largest share in their amino acid composition, which corresponds to other authors [17,21]. Their presence is likely to determine the antiradical ability of the peptides due to their ability to quench unpaired electrons or radicals by supporting protons. Other authors also reported that the presence of these nonpolar amino acids such as Leu and Pro has been correlated to the antioxidant activity [21,38]. This contributes to the radical scavenging activity of peptides due to their special structural characteristics. Chen et al. [39] demonstrated that peptides containing His can act as metal ion chelators, active-oxygen quenchers, and hydroxy radical scavengers. Escudero et al. [17] reported about proline-rich peptide SAGNPN, which showed a high radical scavenging activity. Yet, many synthesized peptides like GGSILI, IAKLE, ALGGA, NVLVG, GLAGA, and NAAKL possessed Leu residues. The presence of Leu possibly contributed to the antioxidant activities of peptides [17,40].

## 3. Materials and Methods

### 3.1. Preparation of Dry-Cured Loins

The pork primal cuts of Polish White Large fatteners (live weight of approximately 120–130 kg) were used in this study. Loins (*M. longissimus thoracis*) were excised at 24 h *post mortem* from half carcasses chilled at 4 °C at a local abattoir (Lublin, Poland). At 48 h post mortem, all loins underwent curing using a surface massage with a mixture of 20 g of sea salt, 9.7 g of curing salt, and 0.3 g of NaNO_3_ per kg of loin. After 24-hour curing at 4 °C, the loins were randomly divided into four experimental batches with three loins each. One of the batches was regarded as a control sample (C). The other three experimental batches were inoculated with *Lactobacillus rhamnosus* (formerly *Lactobacillus casei* ŁOCK 0900) LOCK900 (LOCK), *Lactobacillus acidophilus* Bauer Ł0938 (BAUER), and *Bifidobacterium animalis* ssp. *lactis* BB-12 (BB12), respectively to achieve an initial level of 10^6^–10^7^ CFU/g of meat. The inoculum was prepared at the Department of Food Hygiene and Quality Management (WULSSGGW, Warsaw, Poland) according to the procedure previously described by Wójciak et al. [41]. Subsequently, the loins were hung at 16 ± 1 °C in a disinfected laboratory aging chamber with a relative humidity of between 75% and 80% for 14 days. Then the whole pieces of loins were vacuum-packed in PA/PE (80 μm thick) bags (Wispak, Lublin, Poland) and aged at 4 ± 1 °C for 12 months. Three independent experimental trials were conducted with 12 loins utilized in each trial. After 28, 90, 180, 270, and 360 days of aging, the samples were taken for analysis.

### 3.2. Muscle Proteins Extraction

Water-soluble fraction (WSF) of meat proteins was extracted according to the method described by Molina and Toldrá [42] with modifications suggested by Fadda et al. [43]. To prepare the salt-soluble fraction (SSF), the pellet resulting from the WSF extraction was re-suspended in 0.6 M NaCl in 0.1 M phosphate buffer (pH 6.2) in a ratio of 1:6 and homogenized for 1 min (T25 Basic ULTRA-TURRAX; IKA, Staufen, Germany). The resulting homogenate was deaerated prior to extraction for 18 h at 4 °C. After the centrifugation step at 10,000× *g*, 4 °C for 10 min, the supernatant was filtered through Whatman Filter Paper No. 1. Protein concentration of both fractions was determined by the Biuret method [44] using Liquick Cor-TOTAL PROTEIN 60 kit (Cormay Group, Łomianki, Poland) and bovine serum albumin (BSA) as the standard.

### 3.3. Simulated In Vitro Digestion and Absorption

Muscle protein fractions (WSF and SSF) have been subjected to in vitro digestion using pepsin and pancreatin [16]. Prior to the simulated gastric digestion, protein fractions were adjusted to pH 2.0 with 1 M HCl. Pepsin solution in 6 M HCl (pH 2.0) was added to protein fractions at the ratio of enzyme to substrate of 1:100. The digestion was carried out at 37 °C for 2 h in darkness and under continuous stirring. Afterward, the solution was neutralized to pH 7.0 with 1 M NaOH. For simulated intestinal digestion, pancreatin was added at a 1:50 enzyme to substrate ratio. After incubation at 37 °C for 3 h in darkness with continuous stirring, the enzyme was inactivated by heating at 95 °C for 10 min. Obtained hydrolysates were dialyzed with membrane tubes (molecular weight cut-off 7 kDa; Spectra/Por^®^) against phosphate buffered saline (PBS; pH 7.4; 1:4, *v*/*v*). The absorption process was carried out without light for 1 h at 37 °C.

### 3.4. Primary Amino Groups Content

After each step of in vitro digestion and simulated absorption, the protein degradation products were evaluated by measuring the content of primary amino groups according to the trinitrobenzene sulfonic acid (TNBS) method [45]. The content of primary amino groups (-NH_2_) was expressed as µM/mL of L-leucine amino equivalent based on the calibration curve.

### 3.5. Peptide Identification

Hydrolysates obtained after each step of in vitro digestion and simulated absorption were concentrated in the evaporator and dissolved in 2 mL of 0.01 M HCl prior to chromatographic analysis. The separation of the peptide mixture was done using nanoACQUITY (Waters) liquid chromatography (LC) instruments and Orbitrap Velos Mass Spectrometer (Thermo Electron Corp., San Jose, CA, USA). The peptide mixture was applied to a RP-18 (nanoACQUITY Symmetry C18 Waters 186003545) column using a gradient of acetonitrile (0−35% AcN over 180 min) in the presence of 0.05% formic acid at a flow rate of 250 nL/min. The data was processed by Mascot Distiller and Mascot Search (Matrix science, London, UK) and then compared to the Uniprot database. The search parameters for precursor ions and mass tolerance products were 10 ppm and 0.1. Da. Venn diagrams were applied to analyze the similarity of peptides from each batch.

### 3.6. Determination of Antiradical Activity

#### 3.6.1. In vitro Antiradical Activity

Free radical-scavenging activity of hydrolysates obtained after each step of in vitro digestion and simulated absorption was determined by the ABTS [2,2′-azinobis-(3-ethylbenzothiazoline-6-sulfonic acid)] method according Re et al. [46]. The scavenging activity of the hydrolysates was expressed as the percentage of free radical-scavenging effect using the formula below.

Scavenging [%] = [1 − (As/Ac)] × 100
(1)

where As-absorbance of sample is related to Ac-absorbance of control (ABTS solution). The effective concentration of sample required to scavenge ABTS radical by 50% (IC_50_ value) was obtained by linear regression analysis of the dose-response curve by plotting between percent inhibition and concentration. Nine replicates were performed per sample.

#### 3.6.2. In Silico Antioxidant Activity

The peptide sequences, which were obtained as a result of chromatographic analyses of hydrolysates after in vitro digestion and simulated absorption, were analyzed using the in silico approach. The potential of biological activity was evaluated using tools available in the BIOPEP database i.e., “Profiles of potential biological activity” for distinguishing all peptides with antioxidant properties and “Calculations” to determine the frequency of bioactive fragment occurrence in a protein sequence (A parameter) [47]. The selected peptides were characterized for their amino acids composition, hydrophobicity, and net charge using ProtParm tools [48].

### 3.7. Statistical Analysis

Statistical analysis and comparisons among means were carried out using the SAS statistical software (SAS Institute Inc., Cary, NC, USA). The results were presented as mean ± standard deviation. The data were analyzed by two-way ANOVA. The Tukey’s post hoc test was applied for comparing mean values and differences were considered significant at *p* < 0.05.

## 4. Conclusions

The antiradical activity of WSF and SSF (undigested protein extracts of dry-cured pork loins) and hydrolysates obtained by in vitro digestion have been confirmed. The results suggest that dry-cured pork loin is abundant in natural antioxidants and has the potential to support innate mechanisms to control oxidation processes and can be used to promote human health and food protection. Importantly, the biological activity of these peptides after in vitro digestion at gastrointestinal levels in humans is resistant to the loss of their antiradical bioactivity. It is, however, necessary to establish a correlation between in vitro and in vivo digestion to assess the bioavailability of potential antioxidant peptides.

## Figures and Tables

**Table 1:** Significance levels showed by the experimental factors and their interactions for the antiradical activity of dry-cured loins during long-term aging and in vitro gastrointestinal digestion.

Factor	-NH_2_ (µM/mL)		Antiradical Activity			
			(%)		IC_50_ (µM/mL)	
	WSF	SSF	WSF	SSF	WSF	SSF
**before digestion**						
Treatment (T)	**	**	**	**	**	**
Aging time (S)	**	**	**	**	**	**
TxS	**	**	**	**	**	n.s.
**after pepsin hydrolysis**						
Treatment (T)	**	**	**	**	**	n.s.
Aging time (S)	**	**	**	**	**	**
TxS	**	**	**	**	**	*
**after pepsin/pancreatin hydrolysis**						
Treatment (T)	**	**	**	**	**	**
Aging time (S)	**	**	**	**	**	**
TxS	**	**	**	**	**	**
**after simulated absorption**						
Treatment (T)	**	**	**	**	**	**
Aging time (S)	**	**	**	**	**	**
TxS	**	**	**	**	**	**

-NH_2_, the content of primary amino groups; WSF, water-soluble fraction; SSF, salt-soluble fraction; * *p* < 0.05; ** *p* < 0.01; n.s.: not significant.

**Table 2:** Antiradical activity of protein extracts obtained from undigested dry-cured pork loins (mean ± standard deviation).

Sample	Aging Time (Days)	WSF			SSF		
		-NH_2_ (µM/mL)	Radical Scavenging (%)	IC_50_ (µM/mL)	-NH_2_ (µM/mL)	Radical Scavenging (%)	IC_50_ (µM/mL)
C	28	8.08 ^a,A^ ± 0.04	65.63 ^a,A^ ± 2.14	0.28 ^a,A^ ± 0.01	6.22 ^a,A^ ± 0.83	65.30 ^a,A^ ± 1.87	0.26 ^a,A^ ± 0.03
	90	8.54 ^a,A^ ± 0.17	72.78 ^b,A^ ± 1.08	0.30 ^a,A^ ± 0.01	9.04 ^b,A,B^ ± 0.66	65.60 ^a,A^ ± 2.20	0.20 ^b,A^ ± 0.02
	180	16.10 ^b,A^ ± 1.20	79.14 ^c,A,D^ ± 1.35	0.29 ^a,A^ ± 0.01	7.72 ^a,b,A^ ± 0.82	75.70 ^b,A^ ± 1.44	0.14 ^c,A^ ± 0.01
	270	18.68 ^c,A^ ± 0.86	64.23 ^a,A,B^ ± 1.81	1.02 ^b,A^ ± 0.01	7.27 ^a,b,A^ ± 0.84	59.24 ^c,A^ ± 1.22	0.30 ^d,A^ ± 0.01
	360	29.96 ^d,A^ ± 1.22	59.15 ^d,A^ ± 0.66	2.40 ^c,A^ ± 0.11	11.21 ^c,A^ ± 0.82	51.29 ^d,A^ ± 1.11	0.35 ^e,A^ ± 0.01
LOCK	28	9.02 ^a,A^ ± 0.41	69.94 ^a,B^ ± 2.40	0.23 ^a,B^ ± 0.01	5.57 ^a,A,B^ ± 0.53	62.60 ^a,B^ ± 3.22	0.18 ^a,B^ ± 0.02
	90	9.68 ^a,B^ ± 0.45	75.55 ^b,A,B^ ± 2.26	0.24 ^a,B^ ± 0.01	7.42 ^a,b,A^ ± 1.12	66.96 ^b,A,B^ ± 1.38	0.14 ^b,B^ ± 0.01
	180	17.61 ^b,A^ ± 2.36	78.82 ^b,A^ ± 2.93	0.33 ^a,B^ ± 0.01	7.10 ^a,b,A^ ± 0.76	76.42 ^c,A^ ± 3.31	0.11 ^c,B^ ± 0.01
	270	21.31 ^c,B^ ± 0.05	66.27 ^c,A^ ± 2.28	1.42 ^b,B^ ± 0.08	7.34 ^a,b,A^ ± 0.01	56.54 ^d,B^ ± 1.41	0.35 ^d,B^ ± 0.01
	360	23.44 ^d,B^ ± 0.94	62.48 ^d,B^ ± 1.61	1.68 ^c,B^ ± 0.13	10.78 ^b,B^ ± 0.63	48.82 ^e,B^ ± 0.96	0.28 ^e,B^ ± 0.01
BAUER	28	10.28 ^a,B^ ± 0.95	65.53 ^a,A^ ± 2.38	0.28 ^a,A^ ± 0.02	6.46 ^a,A^ ± 0.18	56.35 ^a,C^ ± 2.53	0.28 ^a,A^ ± 0.03
	90	9.37 ^a,A,B^ ± 0.20	77.53 ^b,B^ ± 2.42	0.27 ^a,C^ ± 0.01	8.90 ^b,A,C^ ± 0.80	66.17 ^b,A,B^ ± 0.39	0.24 ^a,C^ ± 0.01
	180	20.62 ^b,A^ ± 1.83	83.89 ^c,C^ ± 1.86	0.27 ^a,C^ ± 0.01	11.22 ^c,B^ ± 0.29	63.55 ^c,B^ ± 2.31	0.18 ^b,C^ ± 0.05
	270	22.76 ^b,C^ ± 1.21	62.89 ^d,B^ ± 1.13	1.46 ^b,B,C^ ± 0.16	10.78 ^c,B^ ± 0.36	68.68 ^d,C^ ± 1.62	0.35 ^c,B^ ± 0.03
	360	30.91 ^c,A^ ± 0.71	61.52 ^d,A,B^ ± 1.11	1.66 ^b,B^ ± 0.16	12.58 ^c,A^ ± 1.22	43.31 ^e,C^ ± 1.18	0.34 ^c,B^ ± 0.01
BB12	28	10.68 ^a,B^ ± 0.15	64.99 ^a,A^ ± 2.33	0.29 ^a,A^ ± 0.01	4.95 ^a,B^ ± 0.45	60.04 ^a,D^ ± 1.63	0.20 ^a,B^ ± 0.01
	90	10.89 ^a,C^ ± 0.56	76.12 ^b,B^ ± 3.53	0.29 ^a,A^ ± 0.01	10.31 ^b,B,C^ ± 2.62	67.83 ^b,B^ ± 2.70	0.18 ^a,D^ ± 0.01
	180	17.63 ^b,A^ ± 1.30	81.17 ^c,D^ ± 1.10	0.22 ^a,D^ ± 0.01	9.47 ^b,c,B^ ± 0.56	72.31 ^c,C^ ± 1.41	0.13 ^b,A^ ± 0.01
	270	26.26 ^c,D^ ± 0.02	74.12 ^b,C^ ± 1.41	1.79 ^b,C^ ± 0.16	7.04 ^c,A^ ± 0.61	62.50 ^d,D^ ± 1.52	0.26 ^c,A^ ± 0.17
	360	29.07 ^c,A^ ± 2.32	61.42 ^d,A,B^ ± 0.70	1.66 ^b,B^ ± 0.16	10.22 ^b,A,B^ ± 0.62	51.70 ^e,A,B^ ± 2.41	0.25 ^c,A^ ± 0.01

-NH_2_, the content of primary amino groups; WSF, water-soluble fraction; SSF, salt-soluble fraction; C, control sample; LOCK, sample inoculated with *Lactobacillus rhamnosus* LOCK900; BAUER, sample inoculated with *Lactobacillus acidophilus* Bauer Ł0938; BB12, sample inoculated with *Bifidobacterium animalis* ssp. *lactis* BB-12; ^a–e^ Within the same treatment, means followed by a common letter do not differ significantly (*p* < 0.05); ^A–D^ Within the same aging time, means followed by a common letter do not differ significantly (*p* < 0.05).

**Table 3:** The Pearson’s correlation coefficients between ABTS 1/IC_50_ values and the content of protein degradation products.

Fraction	Antiradical Activity	-NH_2_ (µM/mL)
WSF	%	−0.399
	1/IC_50_	−0.833
SSF	%	−0.273
	1/IC_50_	−0.247

-NH_2_, the content of primary amino groups; WSF, water-soluble fraction; SSF, salt-soluble fraction.

**Table 4:** Antiradical activity of protein hydrolysates after gastric digestion (mean ± standard deviation).

Sample	Aging Time (Days)	WSF			SSF		
		-NH_2_ (µM/mL)	Radical Scavenging (%)	IC_50_ (µM/mL)	-NH_2_ (µM/mL)	Radical Scavenging (%)	IC_50_ (µM/mL)
C	28	15.26 ^a,A,B^ ± 1.28	61.22 ^a,A^ ± 1.15	0.66 ± 0.06 ^a,A^	14.74 ^a,A,C^ ± 0.72	57.98 ^a,A,C^ ± 1.50	0.87 ^a,A^ ± 0.05
	90	16.20 ^a,A^ ± 0.49	81.77 ^b,A^ ± 1.77	0.92 ± 0.03 ^b,A^	8.09 ^b,A^ ± 0.54	69.33 ^b,A^ ± 0.76	0.50 ^b,A^ ± 0.01
	180	22.27 ^b,A^ ± 1.16	76.33 ^c,A^ ± 1.68	0.20 ± 0.01 ^c,A,B^	11.44 ^c,A^ ± 0.60	59.47 ^a,A^ ± 0.91	0.13 ^c,A^ ± 0.01
	270	24.32 ^b,A^ ± 1.36	60.27 ^a,A^ ± 0.97	2.02 ± 0.03 ^d,A^	9.61 ^d,A^ ± 0.47	47.67 ^c,A^ ± 1.73	0.76 ^d,A,C^ ± 0.04
	360	28.72 ^c,A,B^ ± 1.97	57.32 ^d,A^ ± 1.01	2.39 ± 0.04 ^e,A^	15.26 ^a,A^ ± 0.50	47.52 ^c,A^ ± 0.97	1.11 ^e,A^ ± 0.07
LOCK	28	16.77 ^a,A^ ± 0.28	61.08 ^a,A^ ± 0.72	0.85 ± 0.05 ^a,B^	14.10 ^a,c,A^ ± 0.02	58.60 ^a,A^ ± 0.80	0.85 ^a,A^ ± 0.06
	90	17.18 ^a,A^ ± 1.38	83.06 ^b,A^ ± 1.43	0.91 ± 0.06 ^a,B^	7.39 ^b,A^ ± 0.76	69.42 ^b,A^ ± 0.98	0.42 ^b,B^ ± 0.03
	180	23.65 ^b,A^ ± 0.85	70.77 ^c,B^ ± 2.04	0.19 ± 0.01 ^b,B^	11.81 ^a,b,c,A,B^ ± 0.78	62.26 ^c,B^ ± 1.34	0.09 ^c,B^ ± 0.05
	270	25.14 ^b,c,A,B^ ± 0.41	59.99 ^a,A^ ± 0.91	1.45 ± 0.06 ^c,B^	7.97 ^b,B^ ± 0.18	52.73 ^d,B^ ± 0.91	0.85 ^a,A,B^ ± 0.03
	360	25.89 ^c,A^ ± 0.72	52.164 ^d,B^ ± 1.78	1.94 ± 0.18 ^d,B^	15.64 ^a,A^ ± 3.09	54.12 ^d,B^ ± 2.67	1.08 ^a,A^ ± 0.26
BAUER	28	14.71 ^a,B^ ± 0.63	59.74 ^a,A^ ± 1.36	0.79 ± 0.05 ^a,B^	12.32 ^a,B^ ± 1.01	54.32 ^a,B^ ± 1.20	0.86 ^a,A^ ± 0.01
	90	19.59 ^b,B^ ± 0.81	83.05 ^b,A^ ± 0.97	0.95 ± 0.03 ^a,C^	7.40 ^b,A^ ± 1.21	70.61 ^b,B^ ± 0.97	0.35 ^b,C^ ± 0.02
	180	27.94 ^c,B^ ± 0.44	76.68 ^c,A^ ± 1.52	0.21 ± 0.01 ^b,A^	13.01 ^a,B^ ± 0.27	55.92 ^c,C^ ± 1.19	0.13 ^c,A^ ± 0.014
	270	29.63 ^d,C^ ± 0.32	54.80 ^d,B^ ± 0.86	2.22 ± 0.11 ^c,C^	10.80 ^a,b,C^ ± 0.41	50.94 ^d,C^ ± 0.80	0.70 ^d,C^ ± 0.04
	360	32.47 ^e,B^ ± 0.76	47.75 ^e,C^ ± 1.83	3.12 ± 0.07 ^d,C^	19.72 ^c,A^ ± 2.70	47.48 ^e,A^ ± 1.10	1.12 ^e,A^ ± 0.11
BB12	28	15.61 ^a,A,B^ ± 0.85	65.38 ^a,B^ ± 1.63	0.82 ± 0.01 ^a,B^	15.83 ^a,C^ ± 0.99	56.27 ^a,C^ ± 1.84	0.90 ^a,A^ ± 0.04
	90	21.31 ^b,C^ ± 0.18	86.51 ^b,B^ ± 0.63	0.83 ± 0.03 ^a,C^	8.74 ^b,A^ ± 1.24	75.18 ^b,C^ ± 0.98	0.44 ^b,B^ ± 0.03
	180	23.10 ^b,c,A^ ± 1.11	73.87 ^c,C^ ± 0.72	0.21 ± 0.01 ^b,A^	11.95 ^c,d,A,B^ ± 0.62	60.09 ^c,A^ ± 0.68	0.12 ^c,A,B^ ± 0.02
	270	26.18 ^c,B^ ± 1.35	57.57 ^d,C^ ± 0.79	1.99 ± 0.07 ^c,A^	7.65 ^b,B^ ± 0.02	49.23 ^d,D^ ± 0.60	0.94 ^a,B^ ± 0.08
	360	29.95 ^d,A,B^ ± 2.39	46.56 ^e,C^ ± 1.22	2.87 ± 0.11 ^d,C^	14.16 ^d,A^ ± 0.48	60.31 ^c,C^ ± 0.70	0.99 ^a,A^ ± 0.04

-NH_2_, the content of primary amino groups; WSF, water-soluble fraction; SSF, salt-soluble fraction; C, control sample; LOCK, sample inoculated with *Lactobacillus rhamnosus* LOCK900; BAUER, sample inoculated with *Lactobacillus acidophilus* Bauer Ł0938; BB12, sample inoculated with *Bifidobacterium animalis* ssp. *lactis* BB-12; ^a–e^ Within the same treatment, means followed by a common letter do not differ significantly (*p* < 0.05);^A–D^ Within the same aging time, means followed by a common letter do not differ significantly (*p* < 0.05).

**Table 5:** Antiradical activity of protein hydrolysates after gastric-intestinal digestion (mean ± standard deviation).

Sample	Aging Time (Days)	WSF			SSF		
		-NH_2_ (µM/mL)	Radical Scavenging (%)	IC_50_ (µM/mL)	-NH_2_ (µM/mL)	Radical Scavenging (%)	IC_50_ (µM/mL)
C	28	21.08 ^a,A,B^ ± 1.24	86.43 ^a,A^ ± 1.00	0.16 ^a,A^ ± 0.01	10.61 ^a,A^ ± 2.39	82.31 ^a,A^ ± 0.64	0.17 ^a,A^ ± 0.01
	90	18.63 ^a,A^ ± 0.42	93.49 ^b,A^ ± 1.73	0.35 ^b,A^ ± 0.02	15.36 ^b,A,B^ ± 1.88	85.71 ^b,A^ ± 0.50	0.28 ^b,A^ ± 0.01
	180	28.88 ^b,A^ ± 1.32	96.01 ^c,A^ ± 0.68	0.17 ^a,A,B^ ± 0.02	12.88 ^c,A^ ± 0.25	87.06 ^c,A^ ± 0.90	0.13 ^c,A^ ± 0.02
	270	18.68 ^a,A^ ± 0.86	94.93 ^c,A^ ± 0.62	0.22 ^a,A^ ± 0.01	7.30 ^d,A^ ± 0.84	95.86 ^d,A^ ± 0.37	0.14 ^a,c,A^ ± 0.01
	360	31.39 ^b,A,B^ ± 2.41	91.73 ^d,A^ ± 0.76	0.51 ^c,A^ ± 0.06	15.87 ^b,A^ ± 2.28	94.84 ^d,A^ ± 1.96	0.14 ^a,c,A,B^ ± 0.01
LOCK	28	20.88 ^a,A,B^ ± 1.30	88.63 ^a,B^ ± 0.72	0.19 ^a,B^ ± 0.02	10.46 ^a,A^ ± 1.49	72.81 ^a,B^ ± 1.91	0.15 ^a,B^ ± 0.01
	90	21.24 ^a,B^ ± 1.27	96.67 ^b,B^ ± 0.69	0.32 ^b,A^ ± 0.01	16.56 ^b,A^ ± 1.45	89.34 ^b,B^ ± 0.74	0.23 ^b,B^ ± 0.01
	180	25.68 ^b,B^ ± 2.01	96.33 ^b,d,A^ ± 0.55	0.19 ^a,A^ ± 0.01	18.81 ^b,B^ ± 2.26	90.05 ^b,B^ ± 1.32	0.11 ^c,A^ ± 0.01
	270	21.31 ^a,B^ ± 0.05	97.83 ^c,B^ ± 0.50	0.21 ^a,A^ ± 0.01	7.34 ^c,A^ ± 0.01	91.01 ^b,c,B^ ± 0.68	0.14 ^a,c,A^ ± 0.01
	360	27.94 ^b,A^ ± 1.10	95.58 ^d,B^ ± 0.66	0.43 ^c,B^ ± 0.01	16.40 ^b,A^ ± 0.50	91.82 ^c,B^ ± 1.19	0.12 ^c,A^ ± 0.01
BAUER	28	19.00 ^a,B^ ± 1.17	85.94 ^a,A^ ± 0.98	0.14 ^a,A,C^ ± 0.01	10.63 ^a,A^ ± 1.17	72.45 ^a,B^ ± 1.03	0.14 ^a,C^ ± 0.01
	90	18.59 ^a,A^ ± 0.14	94.41 ^b,A^ ± 0.38	0.41 ^b,B^ ± 0.02	12.40 ^a,B^ ± 2.07	90.95 ^b,c,C^ ± 0.62	0.20 ^b,C^ ± 0.01
	180	37.16 ^b,C^ ± 2.04	98.06 ^c,B^ ± 0.23	0.13 ^a,B^ ± 0.01	18.12 ^b,B^ ± 2.16	90.14 ^b,B^ ± 1.56	0.12 ^a,c,A^ ± 0.05
	270	22.76 ^c,C^ ± 1.21	95.59 ^d,A^ ± 1.09	0.35 ^c,C^ ± 0.01	10.78 ^a,B^ ± 0.36	92.15 ^cC^ ± 0.54	0.13 ^a,c,A^ ± 0.01
	360	33.28 ^d,B^ ± 1.15	95.71 ^d,B^ ± 1.20	0.47 ^d,A,B^ ± 0.01	19.94 ^b,B^ ± 2.35	88.60 ^d,C^ ± 1.54	0.12 ^c,A,C^ ± 0.01
BB12	28	21.61 ^a,A^ ± 1.35	89.52 ^a,B^ ± 1.44	0.13 ^a,C^ ± 0.02	10.78 ^a,A^ ± 0.45	78.93 ^a,C^ ± 1.28	0.19 ^a,D^ ± 0.01
	90	19.35 ^a,A^ ± 0.09	98.56 ^bC^ ± 0.23	0.32 ^b,A^ ± 0.01	14.87 ^b,A,B^ ± 2.18	92.62 ^b,c,D^ ± 0.85	0.22 ^b,B,C^ ± 0.01
	180	31.80 ^b,A^ ± 0.74	96.95 ^c,C^ ± 0.56	0.13 ^a,B^ ± 0.01	17.71 ^c,B^ ± 0.79	91.86 ^b,C^ ± 0.50	0.11 ^c,A^ ± 0.02
	270	26.26 ^c,D^ ± 0.02	98.08 ^b,c,B^ ± 0.33	0.51 ^c,B^ ± 0.02	7.04 ^d,A^ ± 0.61	93.33 ^c,D^ ± 0.52	0.11 ^c,B^ ± 0.01
	360	28.40 ^c,A^ ± 2.89	94.69 ^d,B^ ± 1.29	0.42 ^d,B^ ± 0.01	18.69 ^c,C^ ± 0.45	93.55 ^c,A,B^ ± 1.54	0.15 ^d,B^ ± 0.01

-NH_2_, the content of primary amino groups; WSF, water-soluble fraction; SSF, salt-soluble fraction; C, control sample; LOCK, sample inoculated with *Lactobacillus rhamnosus* LOCK900; BAUER, sample inoculated with *Lactobacillus acidophilus* Bauer Ł0938; BB12, sample inoculated with *Bifidobacterium animalis* ssp. *lactis* BB-12; ^a–d^ Within the same treatment, means followed by a common letter do not differ significantly (*p* < 0.05); ^A–D^ Within the same aging time, means followed by a common letter do not differ significantly (*p* < 0.05).

**Table 6:** Antiradical activity of protein hydrolysates after simulated adsorption (mean ± standard deviation).

Sample	Aging Time (Days)	WSF			SSF		
		-NH_2_ (µM/mL)	Radical Scavenging (%)	IC_50_ (µM/mL)	-NH_2_ (µM/mL)	Radical Scavenging (%)	IC_50_ (µM/mL)
C	28	2.79 ^a,A^ ± 0.03	89.62 ^a,A^ ± 0.65	0.08 ^a,A^ ± 0.01	1.72 ^a,A,C^ ± 0.24	86.19 ^a,A^ ± 1.12	0.03 ^a,A^ ± 0.01
	90	2.26 ^b,A^ ± 0.21	78.46 ^b,A^ ± 1.08	0.07 ^a,A^ ± 0.01	1.35 ^b,A^ ± 0.19	81.32 ^b,A,B^ ± 2.90	0.03 ^a,A^ ± 0.01
	180	4.29 ^c,A^ ± 0.08	80.17 ^c,A^ ± 0.86	0.03 ^b,A^ ± 0.01	2.66 ^a,A^ ± 0.34	90.49 ^c,A^ ± 1.37	0.02 ^a,A^ ± 0.01
	270	3.74 ^d,A^ ± 0.18	83.68 ^d,A^ ± 0.88	0.15 ^c,A,B^ ± 0.02	1.38 ^b,A^ ± 0.07	85.89 ^a,A^ ± 1.11	0.03 ^a,A^ ± 0.01
	360	4.39 ^c,A^ ± 0.31	73.31 ^e,A^ ± 0.75	0.24 ^d,A^ ± 0.01	1.68 ^b,A^ ± 0.06	66.46 ^d,A^ ± 1.49	0.10 ^b,A^ ± 0.02
LOCK	28	2.55 ^a,B^ ± 0.08	91.47 ^a,B^ ± 0.81	0.08 ^a,A^ ± 0.01	1.52 ^a,d,A,B^ ± 0.18	86.98 ^a,A^ ± 1.24	0.03 ^a,b,A^ ± 0.01
	90	2.85 ^bB^ ± 0.24	84.13 ^bB^ ± 1.93	0.06 ^b,A^ ± 0.01	1.21 ^b,A,B^ ± 0.07	79.95 ^b,A^ ± 1.56	0.03 ^a,A^ ± 0.01
	180	4.04 ^c,d,B^ ± 0.05	76.47 ^c,B^ ± 0.83	0.02 ^c,B^ ± 0.01	2.10 ^c,B^ ± 0.04	86.80 ^a,B^ ± 0.61	0.01 ^b,A^ ± 0.01
	270	4.25 ^c,B^ ± 0.01	84.08 ^b,A^ ± 0.95	0.13 ^d,B^ ± 0.01	1.34 ^a,b,A^ ± 0.18	87.26 ^a,B^ ± 0.77	0.03 ^a,B^ ± 0.01
	360	3.96 ^d,A,B^ ± 0.05	70.92 ^d,B^ ± 0.72	0.23 ^e,A^ ± 0.01	1.66 ^d,A^ ± 0.08	70.12 ^c,B^ ± 0.87	0.10 ^c,A^ ± 0.01
BAUER	28	2.32 ^a,C^ ± 0.08	88.61 ^a,A^ ± 1.88	0.08 ^a,A^ ± 0.01	1.96 ^a,C^ ± 0.31	89.63 ^a,B^ ± 0.81	0.02 ^a,B^ ± 0.01
	90	3.15 ^b,B^ ± 0.34	85.65 ^b,B^ ± 1.28	0.09 ^a,B^ ± 0.01	1.06 ^b,B^ ± 0.01	83.63 ^b,B^ ± 1.07	0.03 ^a,A^ ± 0.01
	180	4.41 ^c,A^ ± 0.17	88.96 ^a,C^ ± 0.44	0.02 ^b,B^ ± 0.01	1.58 ^c,C^ ± 0.06	87.26 ^cB^ ± 0.67	0.02 ^a,B^ ± 0.01
	270	4.55 ^c,C^ ± 0.24	68.39 ^c,B^ ± 1.01	0.15 ^c,A^ ± 0.01	2.06 ^a,B^ ± 0.07	83.22 ^b,C^ ± 0.76	0.03 ^a,C^ ± 0.01
	360	4.36 ^c,A^ ± 0.12	74.98 ^d,C^ ± 1.35	0.17 ^d,B^ ± 0.02	2.03 ^a,A^ ± 0.18	72.40 ^d,C^ ± 0.91	0.11 ^b,A^ ± 0.01
BB12	28	2.46 ^a,B^ ± 0.08	91.74 ^a,B^ ± 1.26	0.07 ^a,A^ ± 0.01	1.23 ^a,B^ ± 0.13	85.92 ^a,A^ ± 0.36	0.03 ^a,A^ ± 0.01
	90	3.03 ^b,B^ ± 0.34	88.30 ^b,C^ ± 1.72	0.090 ^b,B^ ± 0.01	1.24 ^a,A,B^ ± 0.08	71.63 ^b,C^ ± 1.66	0.029 ^a,A^ ± 0.01
	180	3.78 ^c,C^ ± 0.01	80.54 ^c,A^ ± 0.97	0.02 ^c,B^ ± 0.01	1.87 ^b,B,C^ ± 0.39	89.58 ^c,A^ ± 0.96	0.017 ^b,A^ ± 0.01
	270	5.25 ^d,D^ ± 0.01	86.38 ^d,C^ ± 0.66	0.17 ^d,A^ ± 0.01	1.28 ^a,A^ ± 0.05	78.72 ^d,D^ ± 1.09	0.03 ^a,A,C^ ± 0.01
	360	3.46 ^c,B^ ± 0.21	81.05 ^c,D^ ± 1.84	0.14 ^e,C^ ± 0.01	1.76 ^a,b,A^ ± 0.47	68.86 ^e,B^ ± 0.62	0.14 ^c,B^ ± 0.01

-NH_2_, the content of primary amino groups; WSF, water-soluble fraction; SSF, salt-soluble fraction; C, control sample; LOCK, sample inoculated with *Lactobacillus rhamnosus* LOCK900; BAUER, sample inoculated with *Lactobacillus acidophilus* Bauer Ł0938; BB12, sample inoculated with *Bifidobacterium animalis* ssp. *lactis* BB-12; ^a–d^ Within the same treatment, means followed by a common letter do not differ significantly (*p* < 0.05); ^A–D^ Within the same aging time, means followed by a common letter do not differ significantly (*p* < 0.05).

**Table 7:** Peptide sequences with the highest antiradical properties obtained after digestion and simulated adsorption of dry-cured pork loin after 180 days of aging.

Peptide Sequence	Mass	Parental Protein	Protein ID ^1^	Bioactive Fragment Location	*A* Parameter	Hydropathicity (GRAVY)	Charge
I**LKP**LE	711.45	Uncharacterized protein	F1SA53; F1SFX4	(2–4) (2–3) (3–4)	0.5000	0.517	0
L**LKP**IE	711.45	Rab GDP dissociation inhibitor beta	Q6Q7J2	(2–4) (2–3) (3–4)	0.5000	0.517	0
L**LKP**LE	711.45	Kinesin-like protein	F1SDL9	(2–4) (2–3) (3–4)	0.5000	0.400	0
**LKP**DPVA	738.43	Serum albumin	F1RUN2; P08835	(1–3) (1–2) (2–3)	0.4286	−0.114	0
AG**LKP**G**EL**	783.45	Phosphoglycerate mutase	B5KJG2	(3–5) (3–4) (7–8)	0.5000	−0.050	0
AS**LKP**EF	790.42	Triosephosphate isomerase	D0G7F6; Q29371	(3–5) (3–4) (4–5)	0.4286	−0.200	0
TL**LKP**NM	815.46	Fructose-bisphosphate aldolase	F1RJ25; F1SSB5	(3–5) (3–4) (4–5)	0.4286	−0.029	1
I**LKP**LED	826.48	Uncharacterized protein	F1SA53; F1SFX4	(2–4) (2–3) (3–4)	0.4286	−0.057	−1
TL**LKP**NM	831.45	Fructose-bisphosphate aldolase	F1RJ25; F1SSB5	(3–5) (3–4) (4–5)	0.4286	−0.029	1
**HLPHD**PM	845.39	Citrate synthase	F1SLZ4; I3LBB3; P00889	(1–4) (1–2) (3–5)	0.4286	−1.57	0
KN**LHPEL**	849.47	L-lactate dehydrogenase A chain	P00339	(3–4) (5–7) (6–7)	0.4286	−1.157	0
**KD**TQ**LHL**	853.47	Myosin-4	Q9TV62; F1SS61	(1–2) (5–7) (5–6) (6–7)	0.5714	−1.029	0.5
QDTK**LHL**	853.47	Uncharacterized protein	F1RN91	(5–7) (5–6) (6–7)	0.4286	−1.029	0.5
**HLPHD**PM	861.38	Citrate synthase	F1SLZ4; I3LBB3; P00889	(1–4) (1–2) (3–5)	0.4286	−1.057	0
A**LKP**T**KP**M	900.51	Phosphoglycerate mutase	B5KJG2	(2–3) (2–4) (3–4) (6–7)	0.5000	−0.525	2
VD**LKP**D**WG**	928.47	Uncharacterized protein	I3LNG8	(3–5) (3–4) (4–5) (7–8)	0.5000	−0.725	−1
YAG**LKP**G**EL**	946.51	Phosphoglycerate mutase	B5KJG2	(4–6) (4–5) (5–6) (8–9)	0.4444	−0.189	0
**EL**PEH**LKP**	961.52	Glutathione S-transferase P	F1RVN0; P80031	(1–2) (6–8) (6–7) (7–8)	0.6250	−1.212	−0.5
AG**LKP**G**EL**PT	981.55	Phosphoglycerate mutase	B5KJG2	(3–5) (3–4) (4–5) (7–8)	0.4000	−0.270	0
QA**LKP**T**KP**M	1012.57	Phosphoglycerate mutase	B5KJG2	(3–5) (3–4) (4–5) (7–8)	0.4444	−0.856	2
**EL**DQA**LKP**T	1013.54	Phosphoglycerate mutase	B5KJG2	(1–2) (6–8) (6–7)	0.4444	−0.811	−1
YAG**LKP**G**EL**P	1043.57	Phosphoglycerate mutase	B5KJG2	(4–6) (4–5) (5–6) (8–9)	0.4000	−0.330	0
E**TW**PP**LKP**S	1053.55	Glutathione S-transferase P	F1RVN0; P80031	(2–3) (6–8) (6–7) (7–8)	0.4444	−1.200	0
LVNS**PHLKPA**	1074.62	Uncharacterized protein	F1S557	(5–7) (6–7) (7–9) (7–8) (8–9)	0.5000	−0.100	1.5
RYAG**LKP**G**EL**	1102.62	Phosphoglycerate mutase	B5KJG2	(5–7) (5–6) (6–7) (9–10)	0.4000	−0.620	1
**EL**PEH**LKP**F	1108.60	Glutathione S-transferase P	F1RVN0; P80031	(1–2) (5–6) (6–8) (6–7) (7–8)	0.5556	−0.767	−0.5
LDQA**LKP**T**KP**	1109.65	Phosphoglycerate mutase	B5KJG2	(5–7) (5–6) (6–7) (9–10)	0.4000	−0.930	1
DQA**LKP**T**KP**M	1127.60	Phosphoglycerate mutase	B5KJG2	(4–6) (4–5) (5–6) (8–9)	0.4000	−1.120	1
ME**TW**PP**LKP**S	1184.59	Glutathione S-transferase P	F1RVN0; P80031	(3–4) (7–9) (7–8) (8–9)	0.4000	−0.890	0
**EL**DQA**LKP**T**KP**M	1385.72	Phosphoglycerate mutase	B5KJG2	(1–2) (6–8) (6–7) (7–8) (10–11)	0.4176	−0.908	0
**HLHWG**SSDDH	1189.49	Carbonic anhydrase 3	Q5S1S4	(1–3) (1–2) (2–3) (2–4) (4–5)	0.5000	−1.570	−0.5
**HLHWG**SSD**DHG**SE	1462.59	Carbonic anhydrase 3	Q5S1S4	(1–3) (2–3) (1–2) (2–4) (4–5) (9–11)	0.4615	−1.569	−1.5
**HLHWG**SSD**DHG**SEH	1599.65	Carbonic anhydrase 3	Q5S1S4	(1–3) (1–2) (2–3) (2–4) (4–5) (9–11)	0.4286	−1.686	−1

^1^ ID from Uniprot; bold refers to the short fragments with antiradical properties located in peptide sequences.

## References

[1] Laranjo M., Elias M., Fraqueza M.J. (2017). The use of starter cultures in traditional meat products. J. Food Qual..

[2] Ojha K.S., Kerry J.P., Duffy G., Beresford T., Tiwari B.K. (2015). Technological advances for enhancing quality and safety of fermented meat products. Trends Food Sci. Technol..

[3] Kęska P., Stadnik J., Zielińska D., Kołożyn-Krajewska D. (2017). Potential of bacteriocins from LAB to improve microbial quality of dry-cured and fermented meat products. Acta Sci. Pol. Technol. Aliment..

[4] Favaro L., Todorov S.D. (2017). Bacteriocinogenic LAB strains for fermented meat preservation: Perspectives, challenges, and limitations. Probiotics Antimicrob..

[5] Kröckel L., Kongo M. (2013). The role of lactic acid bacteria in safety and flavour development of meat and meat products. Lactic Acid Bacteria-R & D for Food, Health and Livestock Purposes.

[6] De Vuyst L., Falony G., Leroy F. (2008). Probiotics in fermented sausages. Meat Sci..

[7] Kołożyn-Krajewska D., Dolatowski Z.J. (2012). Probiotic meat products and human nutrition. Process Biochem..

[8] Neffe-Skocińska K., Okoń A., Kołożyn-Krajewska D., Dolatowski Z. (2017). Amino acid profile and sensory characteristics of dry fermented pork loins produced with a mixture of probiotic starter cultures. J. Sci. Food Agric..

[9] Wójciak K.M., Libera J., Stasiak D.M., Kołożyn-Krajewska D. (2017). Technological aspect of *Lactobacillus acidophilus* Bauer, *Bifidobacterium animalis* BB-12 and *Lactobacillus rhamnosus* LOCK900 use in dry-fermented pork neck and sausage. J. Food Process. Preserv..

[10] Brown L., Pingitore E.V., Mozzi F., Saavedra L., Villegas J.M., Hebert E.M. (2017). Lactic acid bacteria as cell factories for the generation of bioactive peptides. Protein Pept. Lett..

[11] Pessione E., Cirrincione S. (2016). Bioactive molecules released in food by lactic acid bacteria: Encrypted peptides and biogenic amines. Front. Microbiol..

[12] Arihara K. (2006). Strategies for designing novel functional meat products. Meat Sci..

[13] Minkiewicz P., Darewicz M., Iwaniak A., Sokołowska J., Starowicz P., Bucholska J., Hrynkiewicz M. (2015). Common amino acid subsequences in a universal proteome-Relevance for food science. Int. J. Mol. Sci..

[14] Stadnik J., Kęska P. (2015). Meat and fermented meat products as a source of bioactive peptides. Acta Sci. Pol. Technol. Aliment..

[15] Kęska P., Stadnik J. (2016). Porcine myofibrillar proteins as potential precursors of bioactive peptides-an in silico study. Food Funct..

[16] Escudero E., Mora L., Toldrá F. (2014). Stability of ACE inhibitory ham peptides against heat treatment and in vitro digestion. Food Chem..

[17] Escudero E., Mora L., Fraser P.D., Aristoy M.C., Arihara K., Toldrá F. (2013). Purification and identification of antihypertensive peptides in Spanish dry-cured ham. J. Proteom.

[18] Montoro-García S., Zafrilla-Rentero M.P., Celdrán-de Haro F.M., Piñero-de Armas J.J., Toldrá F., Tejada-Portero L., Abellán-Alemán J. (2017). Effects of dry-cured ham rich in bioactive peptides on cardiovascular health: A randomized controlled trial. J. Funct. Foods.

[19] Kęska P., Stadnik J. (2017). Characteristic of antioxidant activity of dry-cured pork loins inoculated with probiotic strains of LAB. CyTA J. Food.

[20] Li X., Wu X, Huang L. (2009). Correlation between antioxidant activities and phenolic contents of radix *Angelicae sinensis* (Danggui). Molecules.

[21] Chen H.M., Muramoto K., Yamauchi F. (1995). Structural analysis of antioxidative peptides from *Soybean Beta-Conglycinin*. J. Agr. Food Chem..

[22] Klompong V., Benjakul S., Kantachote D., Shahidi F. (2007). Antioxidative activity and functional properties of protein hydrolysate of yellow stripe trevally (*Selaroides leptolepis*) as influenced by the degree of hydrolysis and enzyme type. Food Chem..

[23] You L., Zhao M., Cui C., Zhao H., Yang B. (2009). Effect of degree of hydrolysis on the antioxidant activity of loach (*Misgurnus anguillicaudatus*) protein hydrolysates. Innov. Food Sci. Emerg..

[24] You L., Zhao M., Regenstein J.M., Ren J. (2010). Changes in the antioxidant activity of loach (*Misgurnus anguillicaudatus*) protein hydrolysates during a simulated gastrointestinal digestion. Food Chem..

[25] Samaranayaka A.G., Li-Chan E. (2011). Food-derived peptidic antioxidants: A review of their production, assessment, and potential applications. J. Funct. Foods.

[26] Nalinanon S., Benjakul S., Kishimura H., Shahidi F. (2011). Functionalities and antioxidant properties of protein hydrolysates from the muscle of ornate threadfin bream treated with pepsin from skipjack tuna. Food Chem..

[27] Chang O.K., Ha G.E., Jeong S.G., Seol K.H., Oh M.H., Kim D.W., Ham J.S. (2013). Antioxidant activity of porcine skin gelatin hydrolyzed by pepsin and pancreatin. Korean J. Food Sci. An..

[28] Zhu C.Z., Zhang W.G., Kang Z.L., Zhou G.H., Xu X.L. (2014). Stability of an antioxidant peptide extracted from Jinhua ham. Meat Sci..

[29] Sarmadi B.H., Ismail A. (2010). Antioxidative peptides from food proteins: A review. Peptides.

[30] Paolella S., Falavigna C., Faccini A., Virgili R., Sforza S., Dall’Asta C., Galaverna G. (2015). Effect of dry-cured ham maturation time on simulated gastrointestinal digestion: Characterization of the released peptide fraction. Food Res. Int..

[31] Bauchart C., Morzel M., Chambon C., Mirand P.P., Reynès C., Buffière C., Rémond D. (2007). Peptides reproducibly released by in vivo digestion of beef meat and trout flesh in pigs. Br. J. Nutr..

[32] Escudero E., Sentandreu M.A., Toldra F. (2010). Characterization of peptides released by in vitro digestion of pork meat. J. Agr. Food Chem..

[33] Li L., Liu Y., Zhou G., Xu X., Li C. (2017). Proteome profiles of digested products of commercial meat sources. Front. Nutr..

[34] Chen Y., Kwon S.W., Kim S.C., Zhao Y. (2005). Integrated approach for manual evaluation of peptides identified by searching protein sequence databases with tandem mass spectra. J. Proteome Res..

[35] Power O., Jakeman P., FitzGerald R.J. (2013). Antioxidative peptides: Enzymatic production, in vitro and in vivo antioxidant activity and potential applications of milk-derived antioxidative peptides. Amino Acids.

[36] Ren J., Zhao M., Shi J., Wang J., Jiang Y., Cui C., Xue S.J. (2008). Purification and identification of antioxidant peptides from grass carp muscle hydrolysates by consecutive chromatography and electrospray ionization-mass spectrometry. Food Chem..

[37] Xie Z., Huang J., Xu X., Jin Z. (2008). Antioxidant activity of peptides isolated from alfalfa leaf protein hydrolysate. Food Chem..

[38] Peña-Ramos E.A., Xiong Y.L., Arteaga G.E. (2004). Fractionation and characterisation for antioxidant activity of hydrolysed whey protein. J. Sci. Food Agric..

[39] Chen H.M., Muramoto K., Yamauchi F., Nokihara K. (1996). Antioxidant activity of designed peptides based on the antioxidative peptide isolated from digests of a soybean protein. J. Agr. Food Chem..

[40] Hsu K.C. (2010). Purification of antioxidative peptides prepared from enzymatic hydrolysates of tuna dark muscle by-product. Food Chem..

[41] Wójciak K.M., Dolatowski Z.J., Kołożyn-Krajewska D., Trząskowska M. (2012). The effect of the *Lactobacillus casei* LOCK 0900 probiotic strain on the quality of dry-fermented sausage during chilling storage. J. Food Qual..

[42] Molina I., Toldrá F. (1992). Detection of proteolytic activity in microorganisms isolated from dry-cured ham. J. Food Sci..

[43] Fadda S., Sanz Y., Vignolo G., Aristoy M.C., Oliver G., Toldrá F. (1999). Characterization of muscle sarcoplasmic and myofibrillar protein hydrolysis caused by *Lactobacillus plantarum*. Appl. Environ. Microbiol..

[44] Gornall A.G., Bardawill C.J., David M.M. (1949). Determination of serum proteins by means of the biuret reaction. J. Biol. Chem..

[45] Adler-Nissen J. (1979). Determination of the degree of hydrolysis of food protein hydrolysates by trinitrobenzenesulfonic acid. J. Agric. Food Chem..

[46] Re R., Pellegrini N., Proteggente A., Pannala A., Yang M., Rice-Evans C. (1999). Antioxidant activity applying an improved ABTS radical cation decolorization assay. Free Radic. Biol. Med..

[47] Minkiewicz P., Dziuba J., Iwaniak A., Dziuba M., Darewicz M. (2008). BIOPEP database and other programs for processing bioactive peptide sequences. J. AOAC Int..

[48] ProtParam. https://web.expasy.org/protparam/.

